# Transcatheter real-time MRI guided myocardial chemoablation using acetic acid

**DOI:** 10.1186/1532-429X-18-S1-Q68

**Published:** 2016-01-27

**Authors:** Toby Rogers, Srijoy Mahapatra, Steven Kim, Michael Eckhaus, William Schenke, Jonathan R Mazal, Adrienne E Campbell-Washburn, Merdim Sonmez, Anthony Z Faranesh, Kanishka Ratnayaka, Robert J Lederman

**Affiliations:** 1 grid.94365.3d0000000122975165National Heart Lung and Blood Institute, National Institues of Health, Bethesda, MD USA; 2 St Jude Medical, St Paul, MN USA; 3 grid.94365.3d0000000122975165Division of Veterinary Resources, Division of Intramural Research, National Institutes of Health, Bethesda, MD USA; 4 grid.239560.bDepartment of Cardiology, Children's National Medical Center, Washington, DC USA

## Background

In patients with ischemic cardiomyopathy, radiofrequency ablation for ventricular arrhythmias can have limited efficacy because of the mismatch between lesion depth and substrate thickness, and because radiofrequency-induced edema surrounding the lesion is reversible resulting in only temporary conduction block. We hypothesized that transcatheter needle injection under real-time magnetic resonance imaging (MRI) of caustic agents doped with gadolinium contrast could achieve deep targeted and irreversible myocardial ablation which could be assessed acutely.

## Methods

Caustic agent (ethanol or acetic acid) was injected into the myocardium of 8 swine using MRI-conspicuous needle catheters incorporating loopless antennae for active visualization under real-time MRI. MRI and histological appearances of acute and chronic lesions were compared. An animal model of ischemic cardiomyopathy was created in 10 swine by sub-selective transcoronary ethanol administration into non-contiguous territories. The conductive isthmus between adjacent infarcts was targeted for chemoablation under direct real-time MRI guidance. Baseline and post-chemoablation electroanatomic mapping was performed under X-ray fluoroscopic guidance using a commercial system.

## Results

**Figure 1:**
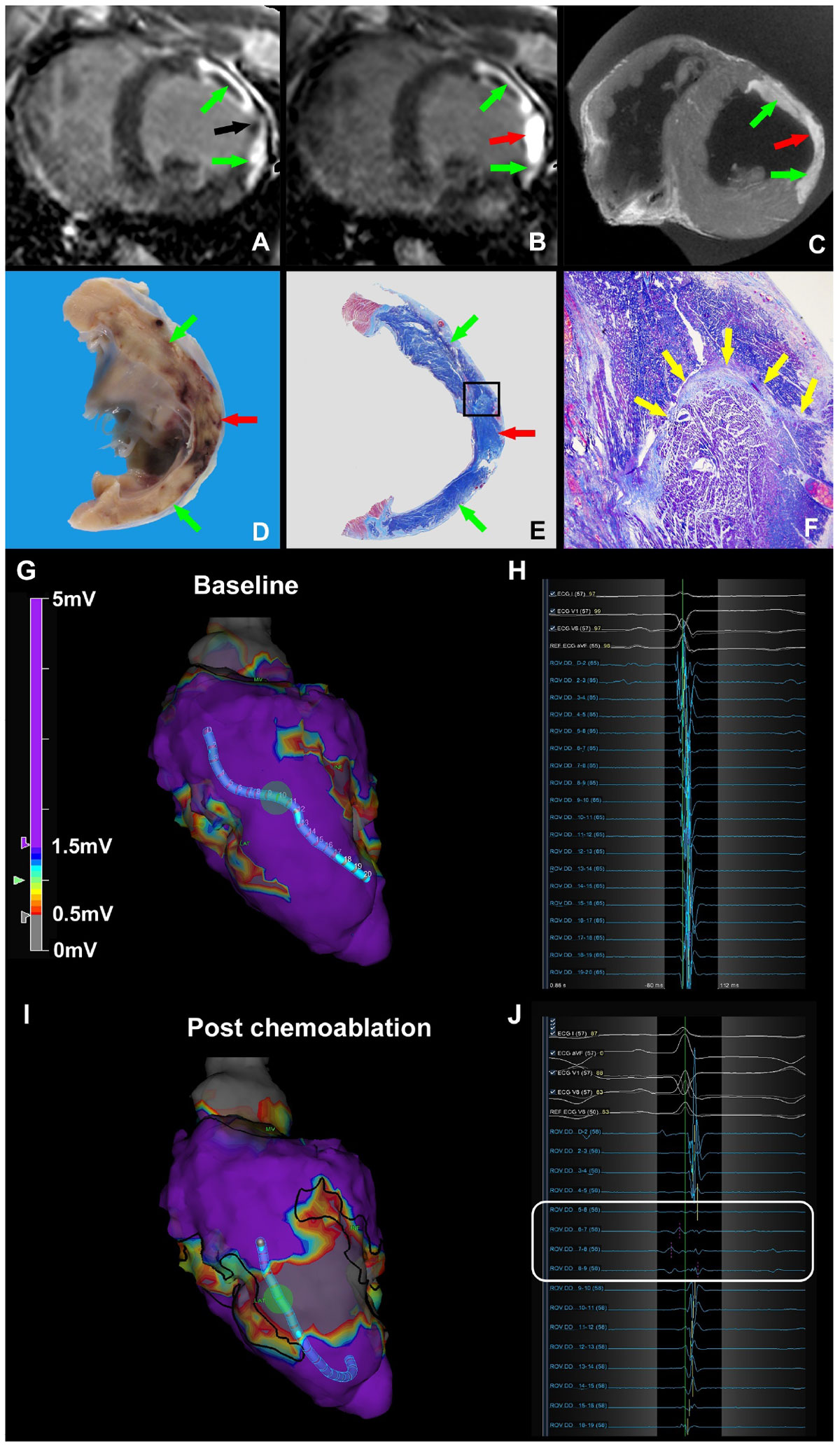
**Real-time MRI guided chemoablation of a conductive isthmus in a porcine model of ischemic cardiomyopathy**.

Ethanol caused stellate lesions with skip areas of normal myocardium, whereas acetic acid caused circumscribed lesions of irreversible necrosis. Acetic acid chemoablation lesions had identical geometry by in vivo and ex-vivo MRI late gadolinium enhancement and histopathology, immediately and after 12(7-17) days. In the animal model of ischemic cardiomyopathy, real-time MRI needle chemoablation successfully eliminated the conductive isthmus immediately by late gadolinium enhancement (Figure 1A-C), histologically (Figure 1D-F), and by electroanatomic voltage mapping (Figure 1G-J).

## Conclusions

Real-time MRI guided myocardial chemoablation with acetic acid is feasible and enables fully transmural substrate-targeted ablation by real-time visualization of arrhythmic substrate and immediate depiction of irreversible ablation lesions. Acetic acid creates more circumscribed and homogeneous lesions than ethanol. Using this technique, we demonstrate anatomical ablation of a conductive isthmus by late gadolinium enhancement and functional abolition of abnormal electrograms in an animal model of ischemic cardiomyopathy. Real-time MRI guided chemoablation could improve efficacy of arrhythmic substrate ablation in the thick ventricular myocardium.

